# Mice Lacking the p43 Mitochondrial T3 Receptor Become Glucose Intolerant and Insulin Resistant during Aging

**DOI:** 10.1371/journal.pone.0075111

**Published:** 2013-09-30

**Authors:** Christelle Bertrand, Emilie Blanchet, Laurence Pessemesse, Jean Sébastien Annicotte, Christine Feillet-Coudray, Béatrice Chabi, Jonathan Levin, Lluis Fajas, Gérard Cabello, Chantal Wrutniak-Cabello, François Casas

**Affiliations:** 1 INRA, Institut National de la Recherche Agronomique, UMR866 Dynamique Musculaire et Métabolisme, Montpellier, France; Université Montpellier 1, Montpellier, France; Université Montpellier 2, Montpellier, France; 2 IGMM, Institut de Génétique Moléculaire de Montpellier, CNRS-UMR5535, Montpellier France; Université Montpellier 1, Montpellier, France; Université Montpellier 2, Montpellier France; Ecole Normale Supérieure de Lyon, France

## Abstract

Thyroid hormones (TH) play an important regulatory role in energy expenditure regulation and are key regulators of mitochondrial activity. We have previously identified a mitochondrial triiodothyronine (T3) receptor (p43) which acts as a mitochondrial transcription factor of the organelle genome, which leads *in vitro* and *in vivo*, to a stimulation of mitochondrial biogenesis. Recently, we generated mice carrying a specific p43 invalidation. At 2 months of age, we reported that p43 depletion in mice induced a major defect in insulin secretion both *in vivo* and in isolated pancreatic islets, and a loss of glucose-stimulated insulin secretion. The present study was designed to determine whether p43 invalidation influences life expectancy and modulates blood glucose and insulin levels as well as glucose tolerance or insulin sensitivity during aging. We report that from 4 months old onwards, mice lacking p43 are leaner than wild-type mice. p43−/− mice also have a moderate reduction of life expectancy compared to wild type. We found no difference in blood glucose levels, excepted at 24 months old where p43−/− mice showed a strong hyperglycemia in fasting conditions compared to controls animals. However, the loss of glucose-stimulated insulin secretion was maintained whatever the age of mice lacking p43. If up to 12 months old, glucose tolerance remained unchanged, beyond this age p43−/− mice became increasingly glucose intolerant. In addition, if up to 12 months old p43 deficient animals were more sensitive to insulin, after this age we observed a loss of this capacity, culminating in 24 months old mice with a decreased sensitivity to the hormone. In conclusion, we demonstrated that during aging the depletion of the mitochondrial T3 receptor p43 in mice progressively induced an increased glycemia in the fasted state, glucose intolerance and an insulin-resistance several features of type-2 diabetes.

## Introduction

Thyroid hormones (TH) play an important regulatory role in energy expenditure regulation and are key regulators of mitochondrial activity. TH acts through nuclear receptors (T3Rs) encoded by the TRα and TRβ genes (NR1A1 and NR1A2 according to nuclear hormone receptor nomenclature) [1], [2]. These receptors are ligand-dependent transcription factors that constitutively bind to specific DNA sequences called thyroid hormone response elements (T3RE) located in the promoter of T3 target genes. We have previously identified in mitochondria truncated forms of the nuclear receptor TRα1, with molecular weights of 43 kDa (p43) and 28 kDa [3], [4]. P43 is a mitochondrial T3 receptor ubiquitously expressed which stimulates mitochondrial transcription and protein synthesis in the presence of T3 [5]. In avian QM7 myoblasts or murine C2C12 cells, p43 overexpression stimulates mitochondrial activity and potentiates terminal differentiation whereas direct inhibition of this pathway induces the reverse changes [6], [7], [8]. *In vivo*, we have shown that p43 overexpression in skeletal muscle increases mitochondrial DNA content, mitochondrial respiration, and induces a shift in metabolic and contractile features of muscle fibers towards a slower and more oxidative phenotype [9]. However, this overexpression also induced a potent oxidative stress leading to skeletal muscle atrophy during aging [10]. Recently, we generated mice carrying a specific p43 invalidation, but still expressing TRα1, TRα2 and other TRα proteins [11], [12]. We reported that p43 depletion in mice decreases mitochondrial DNA replication and respiratory chain activity in skeletal muscle, in association with the induction of a more glycolytic muscle phenotype and a decrease of capillary density. In addition, p43−/− mice displayed a significant increase of muscle mass relatively to control animals and had an improved ability to use lipids. Our findings establish that the p43 mitochondrial receptor strongly affects muscle mass and the metabolic and contractile features of myofibers [12].

TH affect different metabolic aspects of glucose and insulin metabolism. Hypothyroidism is associated with a decrease of a normal glucose-stimulated insulin secretion by the β-cells [13]. While an increase of β-cell mass has been reported in hyperthyroidism [14]. In addition, Ligget and co-workers have observed an increase in insulin secretory rate after T3 administration in rat [15]. In accordance with these effects, TRα and TRβ have been identified in adult islet cells [16]. Interestingly, comparison of the expression of the isoforms in mice shows that TRα predominates at early ages in β-cells, that both TRα and TRβ are equally present from postnatal days 9 to 15, and that TRβ becomes the predominant isoform in adult islets [16]. Recently, we reported that p43 depletion in mice induced a major defect in insulin secretion both *in vivo* and in isolated pancreatic islets, and a loss of glucose-stimulated insulin secretion [11]. In addition, p43−/− mice displayed a better insulin sensitivity in skeletal muscle. Moreover, a high fat/high sucrose diet elicited more severe diabetes and glucose intolerance greater than that recorded in normal animals. We also observed both a decrease in pancreatic islet density and in the maximal activity of two complexes (I and IV) of the respiratory chain in isolated pancreatic islets. These dysfunctions were associated with a down regulation of the expression of the glucose transporter Glut2 and Kir6.2, a key component of the K_ATP_ channel [11]. These conjugated effects of the depletion of p43 on insulin secretion and muscle function lead us to study glucose homeostasis on p43−/− mice during aging.

The present study was designed to determine whether p43 invalidation modulates blood glucose and insulin levels as well as glucose tolerance or insulin sensitivity during aging.

## Materials and Methods

### Animals and Ethics Statement

Male mice were housed and maintained on a 12-hour light/dark cycle (lights on at 7∶00 pm). Food and water were provided *ad libitum*. All animal experiments were performed according to European directives (86/609/CEE and 2010/63/CEE) and approved by the Comité d’Ethique en Matière d’Expérimentation Animale: Région Languedoc-Roussillon (No. 324). Our institution guidelines for the care and use of laboratory animals were observed. Our animal facility is approved by the Departmental Veterinary Services (No. C34-172-10) and our Ministry of Research (No. 4962). P43−/− mice, lacking specifically the mitochondrial T3 receptor p43 were generated in our team as described previously [11]. All the mice used in these studies were back-crossed more than ten times into the C57BL/6 background. We generated our colony by crossing p43−/− mice with WT C57BL/6J breeders, and generated future generations of WT controls. To monitor body weight gain/loss, animals were weighed once a month. According to the European Directive 210-63-EU, mice were observed daily for the general health status and mortality scoring. Any obvious signs of disease, injury and behavioral disorder indicating pain was recorded. If signs persist for more than 48 hours the animal was euthanized by cervical dislocation.

### Quantification of WAT

WT and p43−/− males at 2, 5, 12 and 24 months of age were used for WAT quantification. Subcutaneous WAT (from the inguinal region), epididymal WAT and perirenal WAT were dissected and weighed.

### Metabolic Analyses

Oxygen consumption, carbon dioxide production and respiratory exchange ratio (RER) were measured at 22°C using a Comprehensive Lab Animal Monitoring System (Columbus Instruments, Columbus, OH). Male mice were acclimatized individually in metabolic cages with ad libitum access to standard chow and water for 24-h prior to a 24-h period of automated recordings. Sample air from individual cages was passed through sensors to determine O_2_ and CO_2_ content.

### Glucose and Insulin Tolerance Tests

Following an overnight fast, mice were administrated glucose (2 g/kg) by oral gavage, and blood samples for glucose were collected from the tail vein at the indicated times for the glucose measurement. Insulin tolerance was assessed after a 2 hours fast by intra-peritoneal administration of human insulin (0.75 U/kg) and blood glucose monitoring. Glycemia was measured using a OneTouch Ultra2 glucometer (Lifescan).

### Hormone Assays

The serum from males p43−/− and wild-type was collected to determine insulin levels using an insulin ELISA kit (Mercodia, Sweden).

### Histological Analyses

Immunofluorescence and immunohistochemistry were performed as previously described [17], [18]. Briefly, after antigen retrieval, 5 µm formalin-fixed pancreatic sections were incubated with the indicated antibodies (at 1∶300 for insulin and 1∶100 for glucagon). Immunofluorescence stainings were revealed using an Alexa488-conjugated anti guinea-pig (for insulin, Invitrogen) or Texas Red anti-mouse (for glucagon, Jackson immunoresearch).

For the morphometric analyses, pancreatic sections were scanned using a NanoZoomer (Hamamatsu Photonics, Japan) with a 20× objective. Definiens developer 7.1. software was used to analyze and quantify the pictures for each entire section area. For these studies, pancreas from five p43−/− and WT mice were used.

### Statistical Analyses

All results are presented as means ± SEM, or as percentages. Statistical significances of the differences between groups were evaluated with Student’s t-test.

## Results

### After 4 Months, Mice Lacking p43 were Leaner than Wild-type Mice

**Figure 1:**
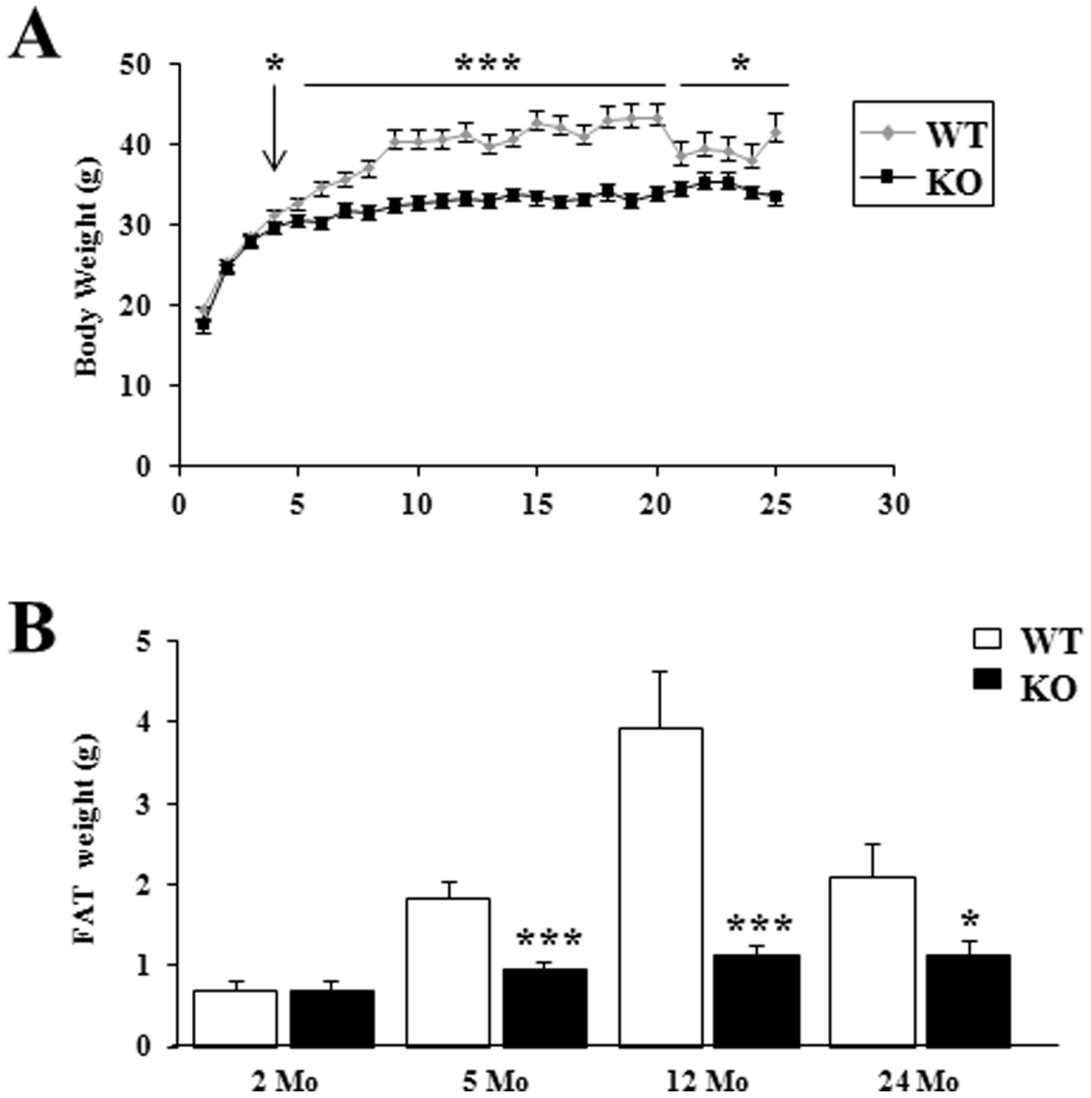
After 4 months, mice lacking p43 were leaner than wild-type mice. (A) p43−/− mice have a lower weight than wild-type mice. Age-dependent changes in body weight in wild-type (WT) and p43−/− mice (KO) were measured each month. Total animals per group ranged from 31 to 14 during aging. (B) p43−/− mice are leaner than wild-type mice. WAT was weighted at 2, 5, 12 and 24 months of age. n = 17 at 5 months, n = 8 at 2, 12, 24 months. Statistical Significance: *p<0.05; ***p<0.001, Student’s *t*-test. Results are expressed as ±s.e.m.

To investigate the effect of the depletion of mitochondrial T3 receptor p43, we started monitoring the body weight of wild type (WT) and p43−/− mice through their entire lifespan. The body weight of WT mice reaches its maximum in 9 months old animals and stabilizes thereafter; the weight of p43−/− animals was significantly lower from 4 to 24 months (Figure 1A). This observation suggested that p43−/− were leaner. White adipose tissue (WAT) weight in male mice was examined at 2, 5, 12 and 24 months. At 2 months of age, WAT weight was similar in WT and p43−/− mice (Figure 1B). Thereafter, if the WAT weight increased markedly in WT it remained low in p43−/− animals (Figure 1B).

### After 5 Months, Mice Lacking p43 had an Increased Metabolism

**Figure 2:**
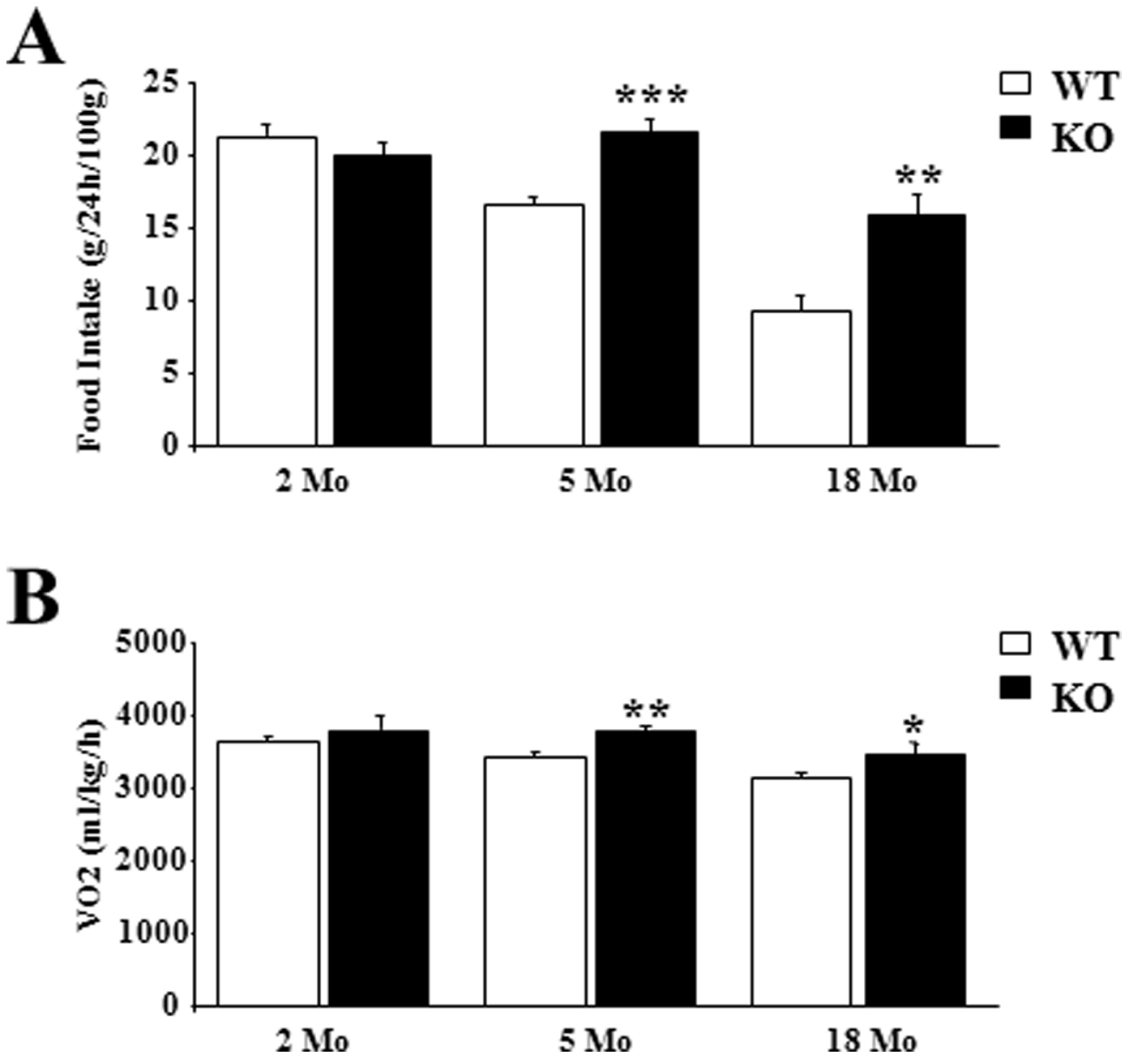
After 5 months, mice lacking p43 had an increased metabolism. (A) Food intake in 2, 5 and 18 month-old male mice (g/24 h/100 g body weight) (n = 8 in each group). (B) Oxygen consumption (ml/kg/h) measured at 22°C using a Comprehensive Lab Animal Monitoring System (Columbus Instruments, Columbus, OH) in 2, 5 and 18 month-old male mice. Male mice were acclimatized individually in metabolic cages with ad libitum access to standard chow and water for 24-h prior to a 24-h period of automated recordings. Sample air from individual cages was passed through sensors to determine O2 and CO2 content. Statistical Significance: *p<0.05; **p<0.01; ***p<0.001, Student’s *t*-test. Results are expressed as ±s.e.m.

This observation suggests that changes in food ingestion or in energy storage occurred in p43−/− animals. To test these possibilities we compared food intake and basal metabolism at 22°C in 2, 5 and 18 months old WT and p43−/− male mice. At 2 months of age, as previously published (Blanchet et al, 2012), food intake was similar in WT and p43−/− mice (Figure 2A). Thereafter, we found that food intake was higher in p43−/− mice than in control ones at 5 (21.5 g/24 h/100 g vs 16.6 g/24 h/100 g, P<0.001) and 18 months (15.9 g/24 h/100 g vs 9.4 g/24 h/100 g, P<0.01) (Figure 2A). If oxygen consumption (ml/kg/h; average 24 h), a measure of energy expenditure, was no different in 2 months old mice, it was about 10% higher in older p43−/− mice compared to WT animals (at 5 months: 3779 ml/kg/h vs 3428 ml/kg/h, P<0.01; at 18 months: 3472 ml/kg/h vs 3151 ml/kg/h, P<0.05) (Figure 2B). This increase was present during both the light and dark cycles (data not shown).

### Blood Glucose Levels were Severely Increased in the Oldest p43−/− Mice

**Figure 3:**
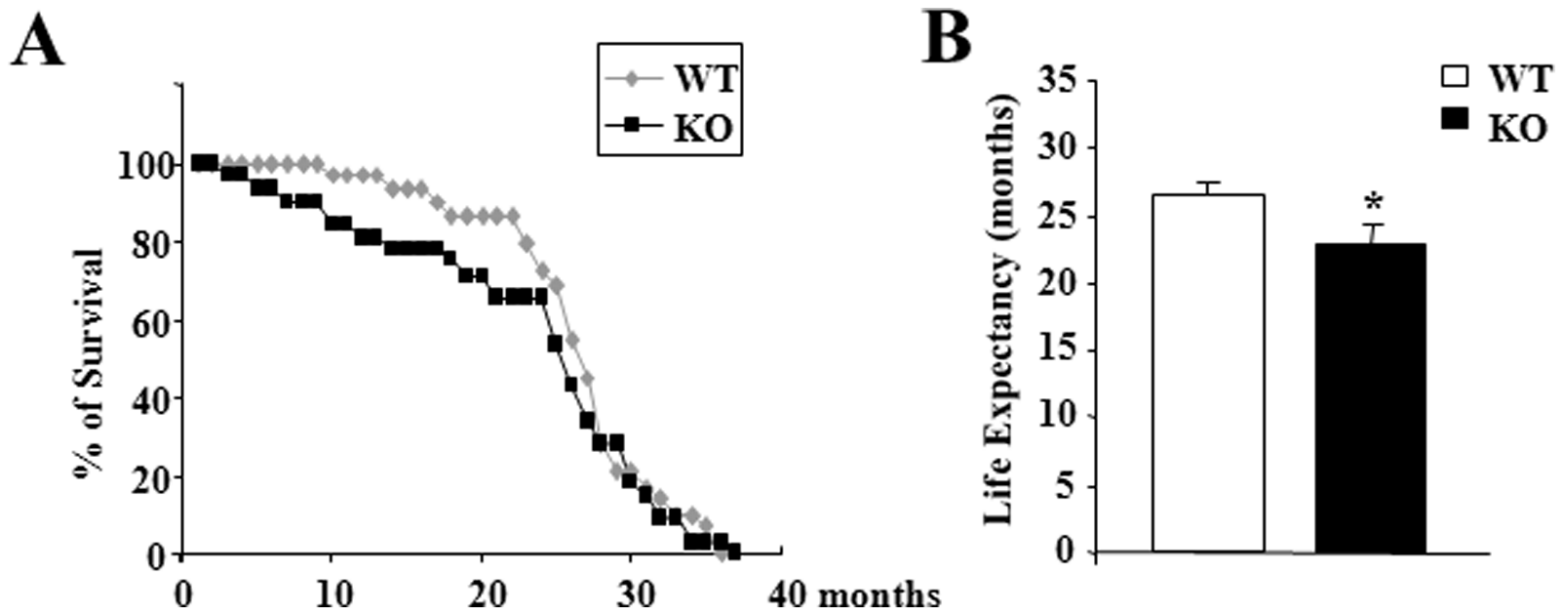
Blood glucose levels were increased in oldest p43−/− mice. (A–E) Blood glucose was measured after an overnight fast (16 hr) or in random-fed states at the indicated age in wild-type (WT) and p43−/− mice (KO) (n = 8 each group). Statistical Significance: ***p<0.001, Student’s *t*-test. Results are expressed as ±s.e.m.

When mice were fed a standard diet, glycemia was not different between wild type and p43−/− mice, after an overnight fasting or in fed conditions at 2, 5, 12 and 18 months (Figure 3A–D). However, we found a strong increase of glycemia in 24 month-old p43−/− mice when compared to controls animals (1.42 g/l vs 1.08 g/l, P<0.001) (Figure 3E).

### Permanent Loss of Glucose-stimulated Insulin Secretion in Mice Lacking p43

**Figure 4:**
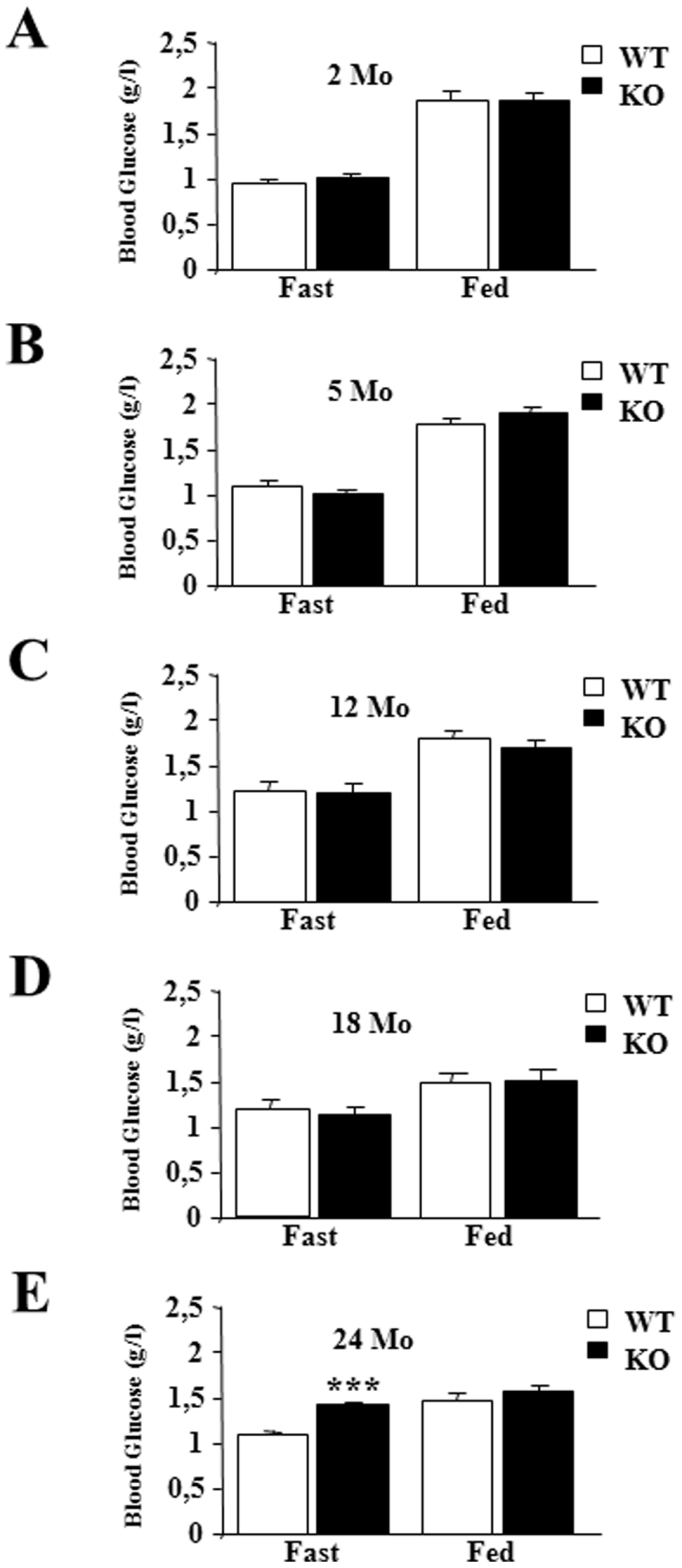
Permanent loss of nutrient-stimulated insulin secretion in mice lacking p43. (A–E) Plasma insulin was measured after an overnight fast (16 hr) or in random-fed states at the indicated age in wild-type (WT) and p43−/− mice (KO) (n = 8 each group). Statistical Significance: *p<0.05; **p<0.01; ***p<0.001, Student’s *t*-test. Results are expressed as ±s.e.m.

We had previously shown in 2 month-old p43−/− mice, a loss of glucose-stimulated insulin secretion [11]. In agreement with this result, plasma insulin levels did not respond to feeding in p43−/− mice whatever the age (Figure 4A–E). In addition, we recorded a strong decrease of plasma insulin levels in 24 month-old p43−/− mice relatively to wild-type animals (Figure 4E).

### P43 Depletion Reduced Pancreatic Islets Density and Impaired their Functionality

**Figure 5:**
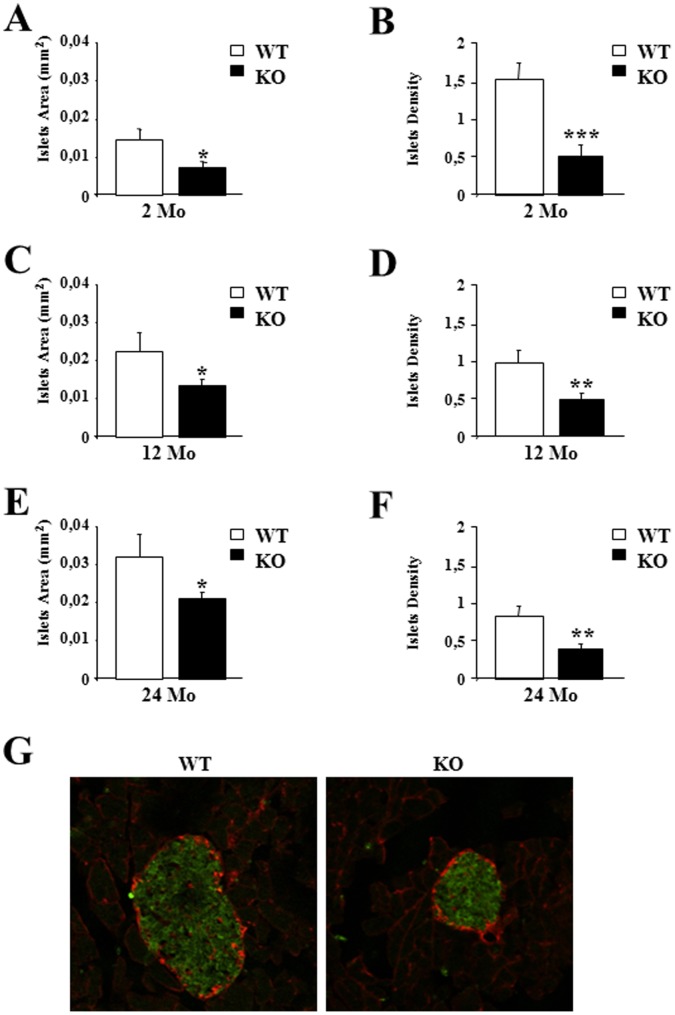
P43 depletion reduced pancreatic islets density and size. (A–F) Quantification of pancreatic islets area and islets density at the indicated age in wild-type (WT) and p43−/− mice (KO) (n = 5 each group). (G) Immunofluorescence staining of serial pancreatic sections showing expression of insulin (green) and glucagon (red). Statistical Significance: *p<0.05; **p<0.01; ***p<0.001, Student’s *t*-test. Results are expressed as ±s.e.m.

Taking into account the permanent loss of insulin response to glycemia induced by p43 deletion and the strong reduction of insulin levels in oldest p43−/− mice, we performed histological analyses using pancreatic sections from control and p43−/− mice at 2, 12 and 24 months. As previously shown at 2 months of age [11], morphological analysis revealed smaller islets in p43−/− animals relatively to control ones associated with a reduced islet density throughout their lifespan (Figure 5A–F). To verify the production of insulin by β-cells in 24 month-old p43−/− mice, we performed immunofluorescence stainings of serial pancreatic sections showing expression of insulin (green) and glucagon (red). As previously shown at 2 months of age [11], we found that the production of both hormones appeared normal in knock-out mice suggesting that the strong decrease of plasma insulin levels recorded previously in the oldest p43−/− mice is more probably linked to a defect of hormone secretion rather than to a deficiency of hormone synthesis (Figure 5G).

### Old p43−/− Mice Displayed a Severe Glucose Intolerance

**Figure 6:**
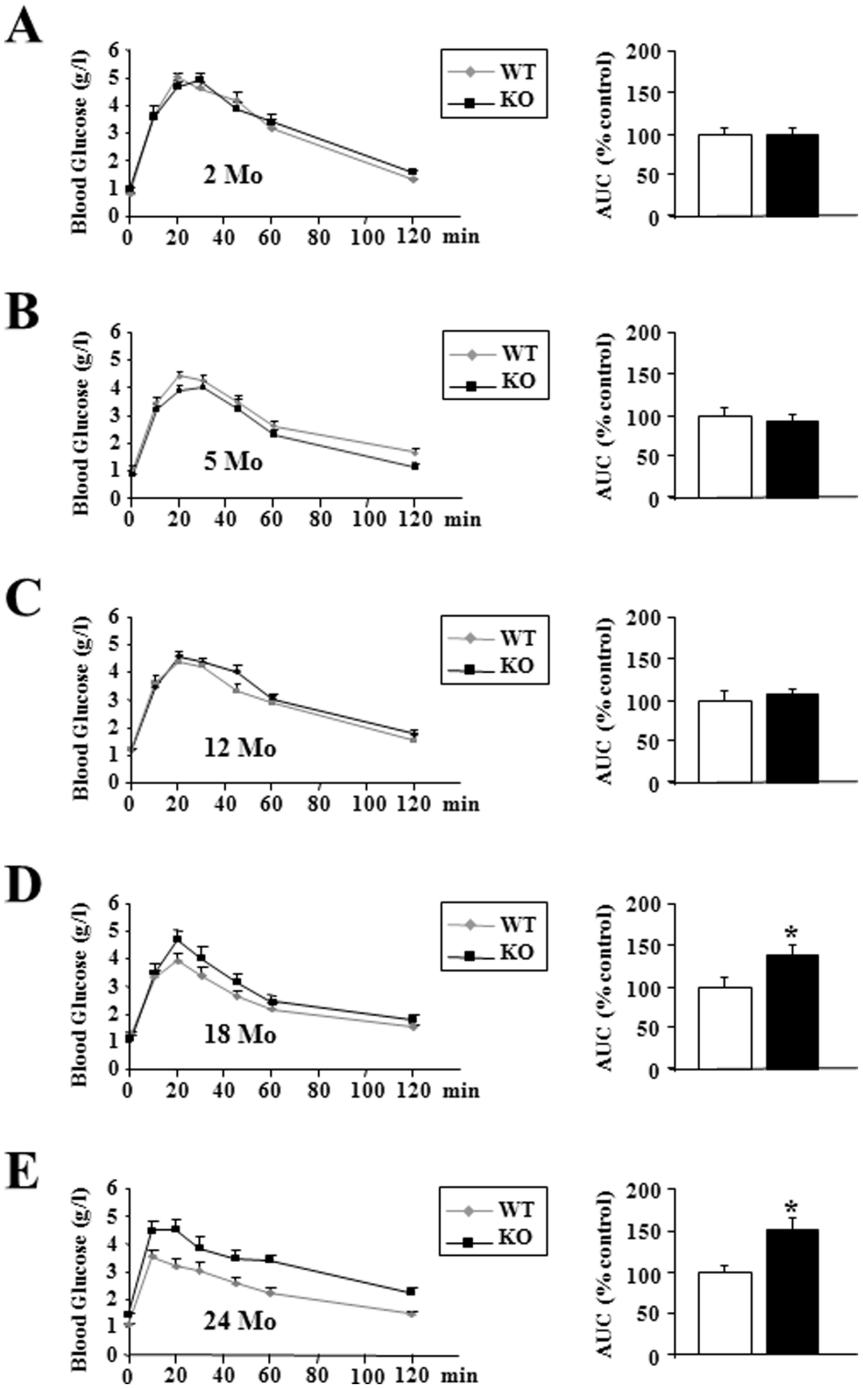
Oldest p43−/− mice displayed a severe glucose intolerance. (A–E) Glucose tolerance test and quantification of the area under the curve at the indicated ages in wild-type (WT) and p43−/− mice (KO) (n = 8 each group). Statistical Significance: *p<0.05, Student’s *t*-test. Results are expressed as ±s.e.m.

Up to 12 months, after a glucose overload, glucose tolerance did not differ between the two groups of animals (Figure 6A–C). However, the oldest p43−/− mice developed a strong glucose intolerance, as confirmed by measurement of the area under the glucose curve (+41% relatively to wild type animals in 18 month-old mice, p<0.05; +51% in 24 month-old mice, p<0.05) (Figure 6D–E).

### p43−/− Mice Became Insulin-resistant during Aging

**Figure 7:**
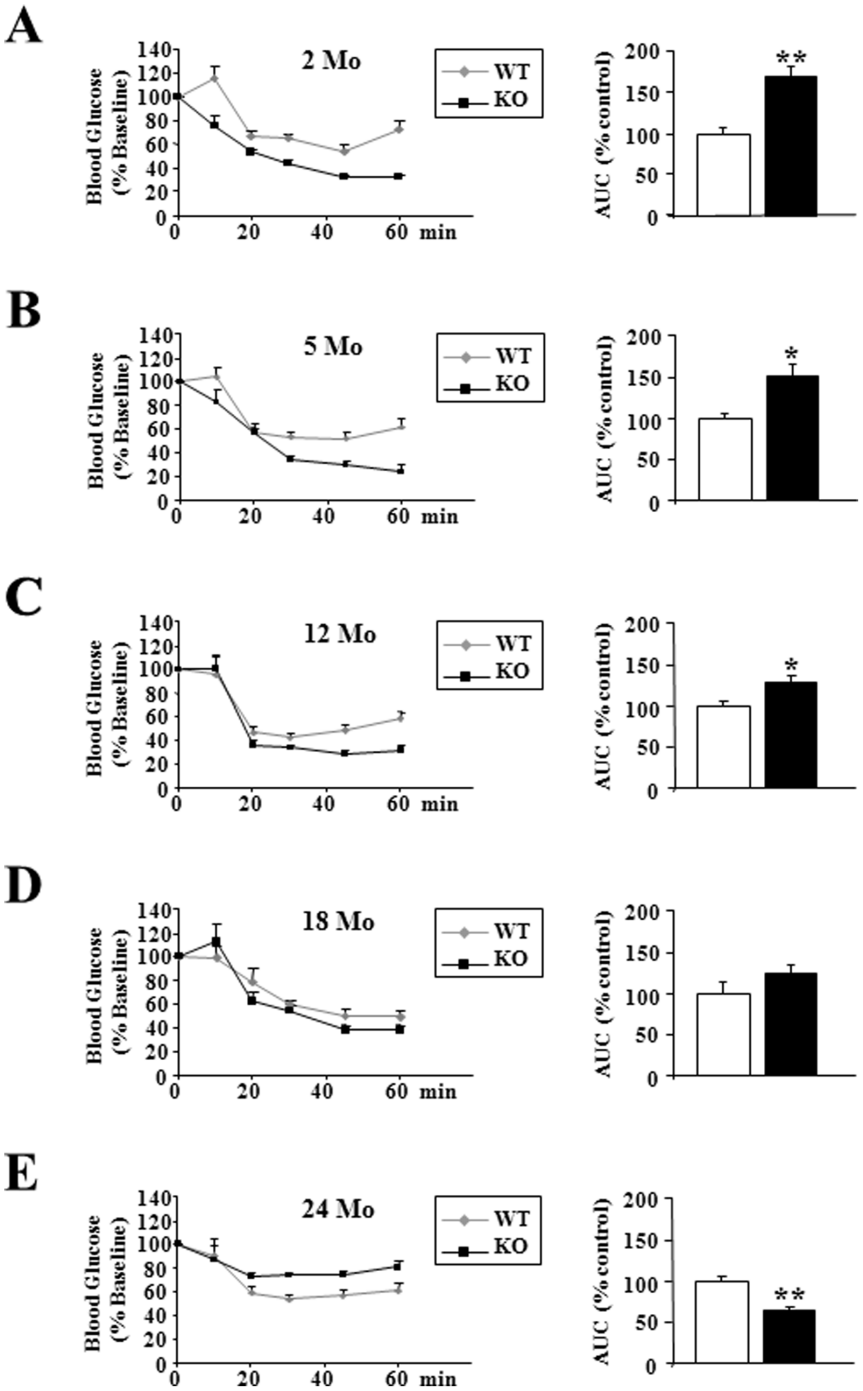
p43−/− mice became insulin-resistant during aging. (A–E) Whole body insulin sensitivity after intra-peritoneal insulin injection (0.75 U/kg) and quantification of the area above the curve at the indicated ages in wild-type (WT) and p43−/− mice (KO) (n = 8 each group). Statistical Significance: *p<0.05; **p<0.01, Student’s *t*-test. Results are expressed as ±s.e.m.

Direct insulin resistance tests were performed by insulin injection, and provided evidence that as previously shown at 2 months of age [11], up to 12 months, p43−/− mice had a better insulin sensitivity, as confirmed by measurement of the area above the glucose curve (+70% relatively to wild type animals in 2 month-old mice, p<0.001; +51% in 5 month-old mice, p<0.05; +29% in 12 month-old mice, p<0.05) (Figure 7A–C). Beyond this age, we observed a loss of this capacity leading to a decreased sensitivity to this hormone in 24 months old p43−/− mice (−36% relatively to wild type animals in 24 month-old mice, p<0.01)(Figure 7E).

## Discussion

Recently, we generated mice carrying a specific p43 invalidation, but which still express TRα1, TRα2 and other TRα proteins [11], [12]. We reported that p43 depletion in mice induced a major defect in insulin secretion and a loss of glucose-stimulated insulin secretion [11]. In addition, this deletion affects muscle mass and the metabolic and contractile features of myofibers [12]. In the present study we attempted to determine whether p43 invalidation modulated blood glucose and insulin levels as well as glucose tolerance and insulin sensitivity during aging at 2, 5, 12, 18 and 24 months.

At 2 months of age, body weight and WAT weight were similar in WT and p43−/− mice. In addition, food intake and basal metabolism were not significantly different. However, after 4 months, p43−/− mice had a lower weight and were leaner than wild-type. The drop in body weight observed between 20 and 21 months in the wild-type mice is linked to a lack of food during a week-end. Curiously, the animals have never recovered their initial weights.

This was at least partly linked to an increase of energy expenditure attested by a higher oxygen and food consumption. These data indicate that a change in metabolism occurs around 4 months in p43−/− mice. Past this age, the observation that p43−/− mice are leaner and display an increase metabolism are in line with a previous study using TRα-0/0 mice lacking all known products of the *Thra* gene including p43 [19], suggesting that this TRα-0/0 phenotype essentially results from p43 absence. However, the phenotype observed in the youngest p43−/− mice indicates that the others proteins encoded by *Thra* gene are able to compensate until 4 months old the p43 deletion and to assure a normal growth and a normal basal metabolism. Altogether this data suggest a complementarity of the TRα proteins in the control of body weight and basal metabolism, with a prominent role of p43 only after the first 4 months of life.

As previously shown in young animals [11], up to 18 months of age plasma glucose levels were similar in wild-type or in mice lacking p43, after overnight fasting or refeeding. In addition, up to 12 months we found that glucose tolerance did not differ between the two groups of animals and that as previously shown [11] p43−/− mice had a better insulin sensitivity. However, in the oldest p43−/− mice, we observed a strong increase of glycemia in fasting condition, the development of glucose intolerance after 18 months and a strong insulin resistance. This set of data indicates that these mice present several features of type-2 diabetes.

In this study, we observed that whatever the age, insulin secretion did not respond in nutrient ingestion in the mice lacking p43. As previously highlighted, this loss of glucose-stimulated insulin secretion in p43−/− mice is probably related to the strong decrease of Kir6.2 and Glut2 expression in pancreatic islets [11]. However, we were unable to check this because it is technically difficult to extract pancreatic islets in aging mice. It is also of note that in the oldest mice we recorded a strong decrease of plasma insulin levels in p43−/− mice in comparison to wild-type animals. This fall in plasma insulin levels could indicate a sharp decrease of the functional β cell mass in these mice. However, the production of the hormone appears normal even at 24 months. Altogether these data suggest that the fall in plasma insulin levels in the oldest mice is most probably related to a further deficit of insulin secretion appears in knockout mice, in addition to those previously described, rather than in a defect of hormone production.

We had previously shown in 2 month-old p43−/− mice a reduction of islet density (−67%). Our analysis performed at 12 and 24 months, indicated that this reduction of islet density did not increase with age. This suggests that the influence of p43 upon islet density initially take place in very young mice and continues throughout their lifespan. Several studies indicate that T3 stimulates pancreatic ductal cells, considered as β-cells precursors [20], [21] and that T3 acts as a mitogenic, pro-survival factor in pancreatic β-cells [21] suggesting that the hormone can be considered a promoting factor for β cell function. Interestingly, Aguayo-Mazzucato and co-workers [16] have recently shown that thyroid hormones can be considered as a physiological regulator of functional maturation of β-cells via its induction of MAFA, a key transcription factor driving the maturation of the insulin secretory response to glucose in neonatal β-cells [22]. In addition, the authors found that TRα predominates at early ages in β-cells, that both TRα and TRβ are equally present from postnatal days 9 to 15, and that TRβ becomes the predominant isoform in adult islets, suggesting that TRα mediates thyroid hormones effects on β-cell during the early postnatal periods [16]. Moreover, Furuya et al [23] have shown that liganded TRα enhances proliferation of pancreatic β-cells. Our results together with these previous studies suggest that *Thra* gene is important during the very early step of β-cell development, important for the appearance of glucose stimulated insulin secretion and determination of β-cell mass. These effects of thyroid hormones on pancreatic β-cells can be mediated by p43 at mitochondrial level and by TRα1 at the nuclear level. In the nucleus, Aguayo-Mazzucato and co-workers [16] have shown that thyroid hormones receptor induces MAFA expression through a direct interaction with the *Mafa* promoter. Interestingly, MAFA could also be an indirect target of p43. Indeed, this hypothesis is supported by our previous data indicating that p43 alters ROS production [24], which are known to regulate MAFA protein stability [25]. Last, the observation that the pancreatic phenotype does not worsen with age in p43−/− mice is in accordance with the fact that TRα is weakly expressed in adult mice. This suggests that the metabolic changes occurring with aging might result from other dysfunctions in particular at the muscular level.

Comparison of the phenotypes observed in p43−/− mice or in TRα deficient mice, indicated that in both cases mice were leaner (after 4 months for p43−/− mice) and manifested greater whole-body insulin sensitivity than wild-type animals at least in the young animals [19], [26], [27]. However, a loss of glucose-stimulated insulin secretion has been reported neither in mice TRα-0/0 [27] nor in a dominant-negative TRα mouse model (R384C) [26]. In addition, our mice lacking p43 are not protected from high-fat-diet induced obesity [11] in contrast to TRα0/0 or R384C mice models. These observations point out the respective influences of the nuclear and mitochondrial T3 pathways demonstrating either similarities (energy expenditure, leanness after 4 months for p43−/− mice) or differences (loss of glucose-stimulated insulin secretion and high-fat-diet induced obesity), thus indicating that these two pathways are complementary and allow a fine-tuning of the thyroid hormones effects. This set of studies also indicates the metabolic functions of the proteins encoded by the *Thra* gene.

Type-2 diabetes mellitus (T2D) is a prevalent disorder of glucose homeostasis resulting from an imbalance between insulin secretion by pancreatic β cells and the sensitivity of peripheral tissues to insulin [28]. In this study, we have demonstrated that the deletion of p43 induced during aging insulin resistance but not obesity. Our results are consistent with previous [29] data indicating that mitochondria play a critical role in two prominent features of T2D: insulin resistance and pancreatic β-cell dysfunction. These data also bring evidence that p43 is a pro-survival factor for β-cells and preserves a normal glucose sensing. Therefore, thyroid hormone through its mitochondrial receptor p43 may counteract diabetes development during aging. In conclusion, our work clearly underlines that deletion or mutation in *THRa* gene affecting p43 expression or function, could induce an increase glycemia, glucose intolerance and an insulin resistance several features of type-2 diabetes in patients.
