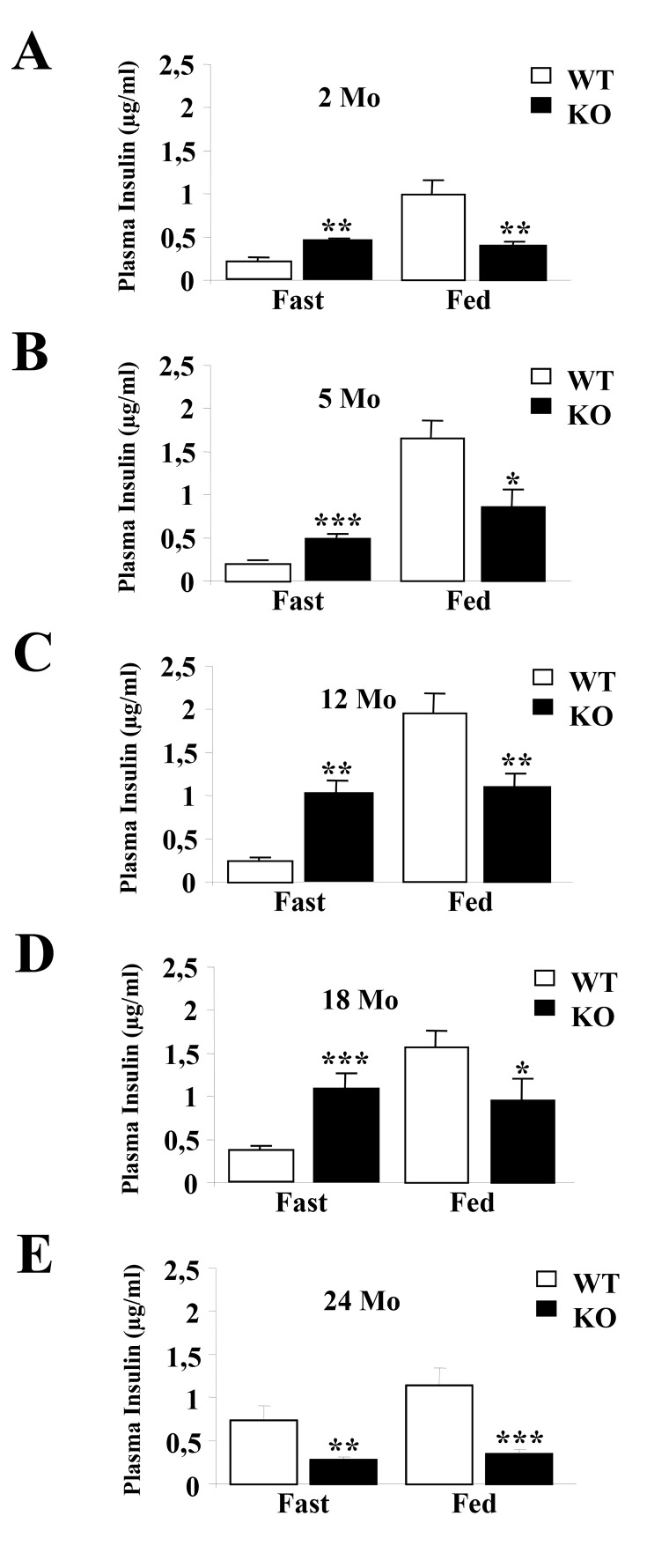# Correction: Mice Lacking the p43 Mitochondrial T3 Receptor Become Glucose Intolerant and Insulin Resistant during Aging

**DOI:** 10.1371/annotation/844c2e7b-2f5f-4982-85d2-b82debdeffb1

**Published:** 2014-01-09

**Authors:** Christelle Bertrand, Emilie Blanchet, Laurence Pessemesse, Jean Sébastien Annicotte, Christine Feillet-Coudray, Béatrice Chabi, Jonathan Levin, Lluis Fajas, Gérard Cabello, Chantal Wrutniak-Cabello, François Casas

Figures 3 and 4 were incorrect. Correct versions of the figures are available below.

Figure 3: 

**Figure:**
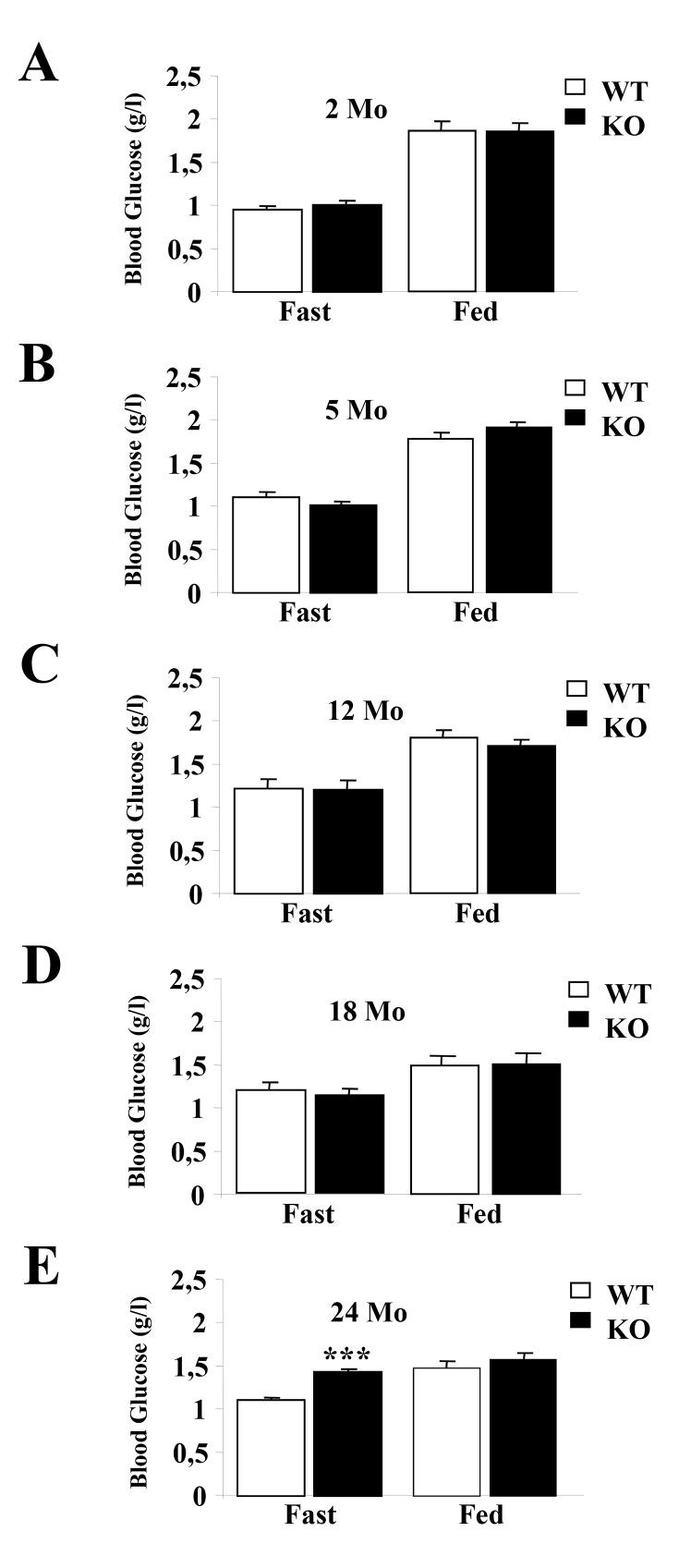


Figure 4: 

**Figure:**